# Supplementary material for: The interactome of intact mitochondria by cross-linking mass spectrometry provides evidence for coexisting respiratory supercomplexes
Source: Mol Cell Proteomics. 2017 Dec 8;17(2):216–32. doi: 10.1074/mcp.RA117.000470 (PMC5795388; doi:10.1074/mcp.RA117.000470)
Supplement: Supplemental Data [file supp_RA117.000470_133922_0_supp_23978_fzffwf.zip › spectra_annotation/mito_DR_spectra_annotation/330-1-14-1-6-1.pdf]

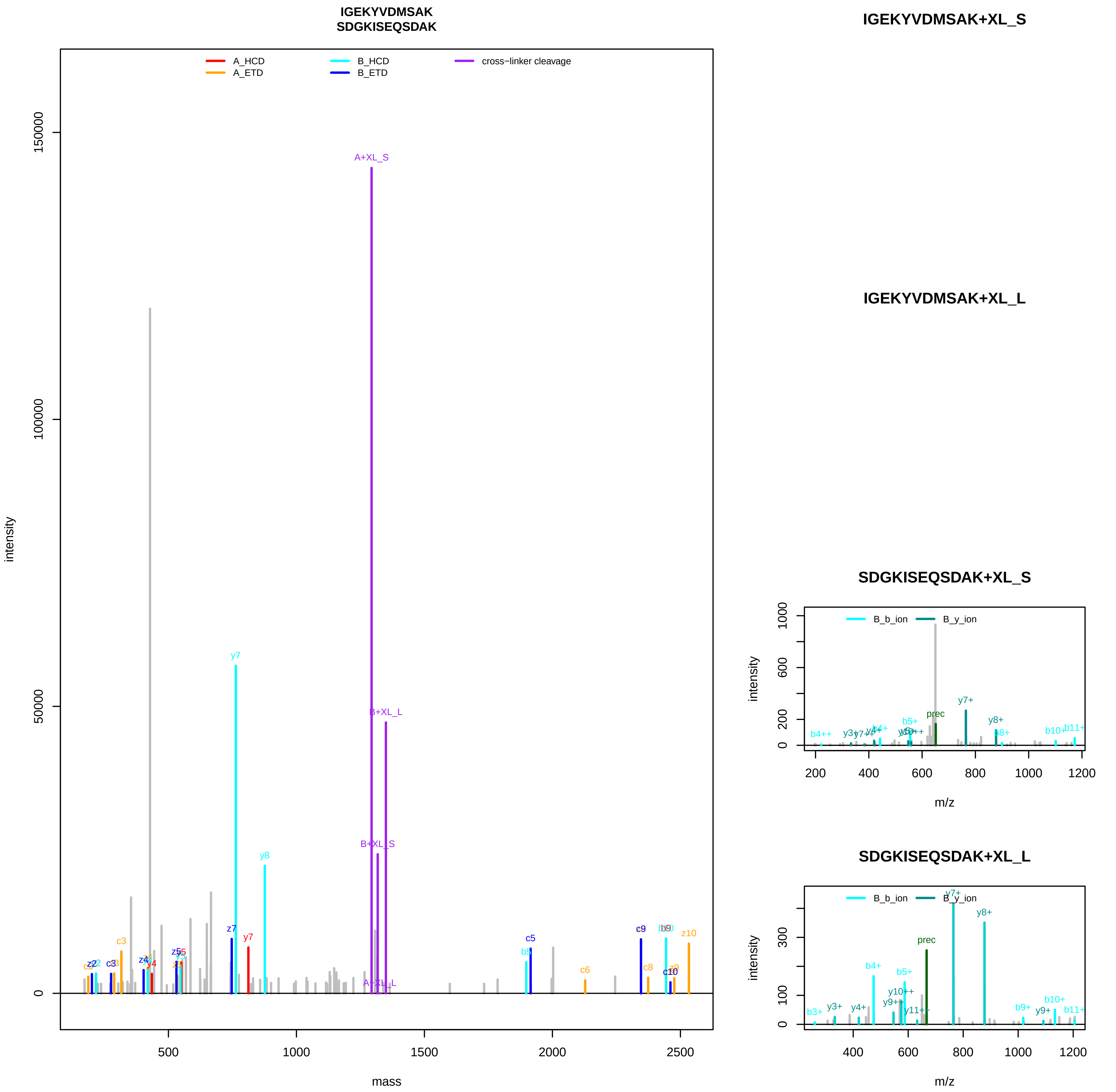
IGEKYVDMSAK
SDGKISEQSDAK
A_HCD
A_ETD
B_HCD
B_ETD
cross−linker cleavage
A+XL_S
B+XL_L
B+XL_S
A+XL_L
y7
y8
z7
y7
c3
c5
b5
c6
c8
c9
c10
b9
z9
z10
y4
0
50000
100000
150000
intensity
500
1000
1500
2000
2500
mass
IGEKYVDMSAK+XL_S
IGEKYVDMSAK+XL_L
SDGKISEQSDAK+XL_S
B_b_ion
B_y_ion
prec
y7+
y8+
b5+
b10+
b11+
0
200
600
1000
intensity
200
400
600
800
1000
1200
m/z
SDGKISEQSDAK+XL_L
B_b_ion
B_y_ion
y7+
y8+
prec
b4+
b5+
b3+
y3+
y4+
b9+
b10+
b11+
0
100
300
intensity
400
600
800
1000
1200
m/z
